# Water energy food nexus model: an integrated aqua-agriculture system to produce tilapia and sweet basil using desalinated water

**DOI:** 10.1007/s11356-022-23240-0

**Published:** 2022-09-30

**Authors:** Hani Sewilam, Fahad Kimera, Peter Nasr

**Affiliations:** 1 Center for Applied Research On the Environment and Sustainability (CARES), School of Science and Engineering, The American University in Cairo, AUC Avenue, P.O. Box 74, New Cairo, 11835 Egypt; 2 UNESCO Chair in Hydrological Changes and Water Resources Management, RWTH Aachen University, Aachen, Germany

**Keywords:** WEF nexus, Organic agriculture, Desalination, Integrated aquaculture, Basil, Linalool

## Abstract

Under the framework of Water-Energy-Food Nexus, this study investigates the integration of solar-powered desalination with aquaculture and agriculture production systems to grow crops. Brackish water desalination is performed using reverse osmosis (RO), the permeate is directed to an aquaculture unit, and the fish effluent is used as irrigation water for crops. The experiment followed a split-plot design with three main variants: a full irrigation schedule for the basil plants with fish effluents as fertilizers, irrigation as fertigation with chemical fertilizers, and a mixed treatment of effluents and chemical fertilizers at 50% application each. Each treatment was further sprayed with or without foliar nutrient application. RO results gave a permeate recovery of 22%, and a salt rejection of 98.7%. In the aquaculture unit, tilapia harvest weight was 0.458 kg with a survival rate of 97.7% and harvest yield of 25 kg/ m^3^ of water. Effluent treatment exhibited the highest branching and plant height irrespective of the cut number, reaching 17.7 branches and 62-cm height. The effluent treatment under foliar application recorded the highest fresh and dry weights of basil with 14.7 ton/ha and 4.7 ton/ha, respectively. Effluent irrigation plus foliar application recorded basil yield of 5 kg/m^3^ effluent water. The dominant essential oil elements were linalool (55.5–61.6%), tau.-cadinol (5.3–8.3%), eucalyptol (5.4–7.7%), eugenol (2–6.8%), and (Z,E)-.alpha.-farnesene (3–5.2%). The study is among the very few integrated systems and, in particular, the current study is the first-time investigation of an integrated desalination, aquaculture, and agriculture production using renewable energy. Therefore, results suggest that integrating desalination, aquaculture, and agriculture could be a potential solution for the global water, energy, and food challenges.

## Introduction 

The world population is rapidly increasing, hitting 7.8 billion as of 2021 (UNFPA 2021). It is predicted that by 2050, the world population will reach 9.6 billion (UN, DESA 2019), hence posing a significant threat to global food security. This population explosion will pressure the declining natural resources such as arable land, renewable energies, and available freshwater (UNICEF 2019). Therefore, it is imperative to investigate for renewable and non-conventional resources such as solar energy and saline water to sustainably produce agricultural products and nutritious food.

Water, energy, and food are limited resources with interlinked and dependent challenges. A more integrated approach to solving these challenges is crucial for any country’s sustainable development. Dealing with the three issues separately is still a common ideology. Integrated studies of these different yet inseparable challenges into one coherent and interdisciplinary endeavor is key to producing lasting solutions towards building a sustainable economy (Daher and Mohtar 2015). The United States Department of Energy illustrates that the Water, Energy, Food Nexus (WEF Nexus) is ideal for any nation’s typical development problems (2014). The WEF Nexus has been recently developed as a thoughtful insight for describing the complicated and interconnected nature of earth’s primary resources on which humankind rely on to attain different social, economic, and environmental goals (Kurian 2017). Because of the interdependencies of the three resources of water, energy, and food, WEF Nexus evolved as a system-based approach to broaden the knowledge regarding sustainable methodologies for managing the three resources (Daher and Mohtar 2015; Bryden 2017). This concept has a significant advantage: it describes both the complex and the dynamic inter-related nature of the global resource system, aiming to balance goals and interests without compromising the quality of the natural ecosystem (Daher and Mohtar 2015).

It is inevitable to focus on research activities related to water, energy, and food while considering their interconnectedness to develop strong agriculture solutions. Many untapped non-conventional solutions are needed to bridge the gap between water scarcity and energy consumption for food production (Kurian 2017). Freshwater availability directly affects food production. The Food and Agriculture Organization estimates 70–95% of global freshwater withdraws are consumed by agriculture (Jury and Vaux 2005; FAO 2017a). For example, irrigation water represents 80% of the total water used in the MENA region, where agricultural water use rose from about 165 BCM in 1995 to more than 218 BCM in 2013, a 32% increase in 18 years (Bryden 2017).

Furthermore, although the Arab region contains 6.3% of the world’s population, it has access to only 1.4% of the world’s renewable freshwater (World Bank 2017). It is expected that by the year 2050, two-thirds of Arab countries could have less than 200 m^3^/person/year of renewable water resources (Zafar 2021).

Thus, one possible water management solution is desalination, which creates new water resources for various water uses and among them is to irrigate high-value crops (Sewilam and Nasr 2017). Nowadays, membrane-based desalination, such as reverse osmosis (RO), is increasingly considered a reliable option for providing water supplies to augment available water sources (Cath et al. 2006; Zhao et al. 2012). RO is increasingly used, not only because of its competitive cost but also due to its continuously improved membranes (Lenntech 2014). Energy requirements for desalination, including RO, can be met through renewable energy resources, such as wind and solar energies. Utilizing renewable energy, particularly solar energy, is especially promising to the Arab Region, which enjoys a huge solar energy potential due to its geographic location (Gnaneswar 2018).

Developing sustainable agricultural solutions on commercial levels is paramount to food security, mainly in developing countries: aquaculture, the farming of aquatic organisms, including fish, mollusks, crustaceans, and aquatic plants. The aquaculture system has already demonstrated its significant role in bridging the global food gap, with its production growing at 7.5% per year since 1970 (FAO 2017b).

Integrated Aqua-Agriculture (IAA) is a technology that utilizes an unconventional approach to fish and crop production (Fernando and Halwart 2000; FAO 2019). Wastewater from the fish production unit is utilized as both irrigation water and a fertilizer solution to provide plants with nutrients, thus enhancing plants’ vegetative growth and fruit production. IAA system involves irrigating plants with fish effluents to maximize crop and fish production while promoting limited water use and fertilizer applications (Edmondson et al. 2019). Previous studies have shown a significant increase in water use efficiency and crop yield when plants are irrigated with fish effluents (Schneider et al. 2005; Dey et al. 2010; Mariscal-Lagarda et al. 2012). The IAA system’s advantage is that it can utilize the linkages and the synergies between the different agro-production activities such as fish, livestock, vegetables, and other high-value crops (Saha et al. 2016; Kimera et al. 2021a). The system demonstrates promising semi-commercial food production results and could provide new agricultural entrepreneurship and investment opportunities (Prein 2002; Abdul-Rahman et al. 2011; Murshed-E-Jahan and Pemsl 2011; Kimera et al. 2021b).

It is a preeminent step to select the most suitable plants and fish species with a high commercial value and suitable use in an IAA system. There is a substantial current global demand for medicinal plants, and the public is gradually accepting them for their significant importance and economic value (Peter and Nirmal Babu 2012; Jamshidi-Kia et al. 2018). Using medicinal plants in numerous health maintenance programs has become an international phenomenon, and the shortage of this vital source will have many implications on both food and traditional health care practices (Kandari et al. 2011; Kurian 2012; Peter and Nirmal Babu 2012). On the other hand, tilapia is considered one of the most hardy, reliable fish, grows rapidly, and possesses strong immune systems with ready market.

Basil (*Ocimum* sp.) is a fast-growing annual plant cultivated in many commercial farming production systems such as hydroponics and aquaponics (Aboutalebi et al. 2013; Mangmang et al. 2016; Saha et al. 2016; Wilson et al. 2017). The major producing countries of this culinary herb are Italy, Spain, France, Egypt, Mexico, Hungary, Germany, and Canada (Walters and Currey 2015; Ferrarezi and Bailey 2019; Sabry et al. 2019). Sweet basil, an antibacterial, antifungal agent, is mainly grown for its fresh and dry leaves and essential oil (EO) which are essentially used in culinary, medicinal, food, and perfumery industry to cure fever, inflammation, and constipation, and is also used in the production of fragrances and flavorings (Chalchat and Özcan 2008; Gajula et al. 2009; Ekren et al. 2012; Ali and Setzer 2014; Ahmed et al. 2014a; Pandey et al. 2016; Alemu et al. 2018). Its EO constitutes of various chemical components such as Linalool, 1,8, cineol, eugenol, methyl cinnamate, camphor, methyl eugenol, methyl chavicol, β-elemene, β-ocimene, camphene, carvacrol, α-bergamotene, α-cadinol, and geranial (Klimánková et al. 2008; Svecová and Neugebauerová 2010). Some studies have been conducted to investigate the productivity of sweet basil in soilless farming like aquaponics and hydroponics. However, there are no or very little cited research on using integrated systems like IAA and desalination for sweet basil production (Nelson and Pade 2008; Gürbüz et al. 2009a; Sharafzadeh and Omid 2011; Yang and Kim 2019).

**Fig. 1:**
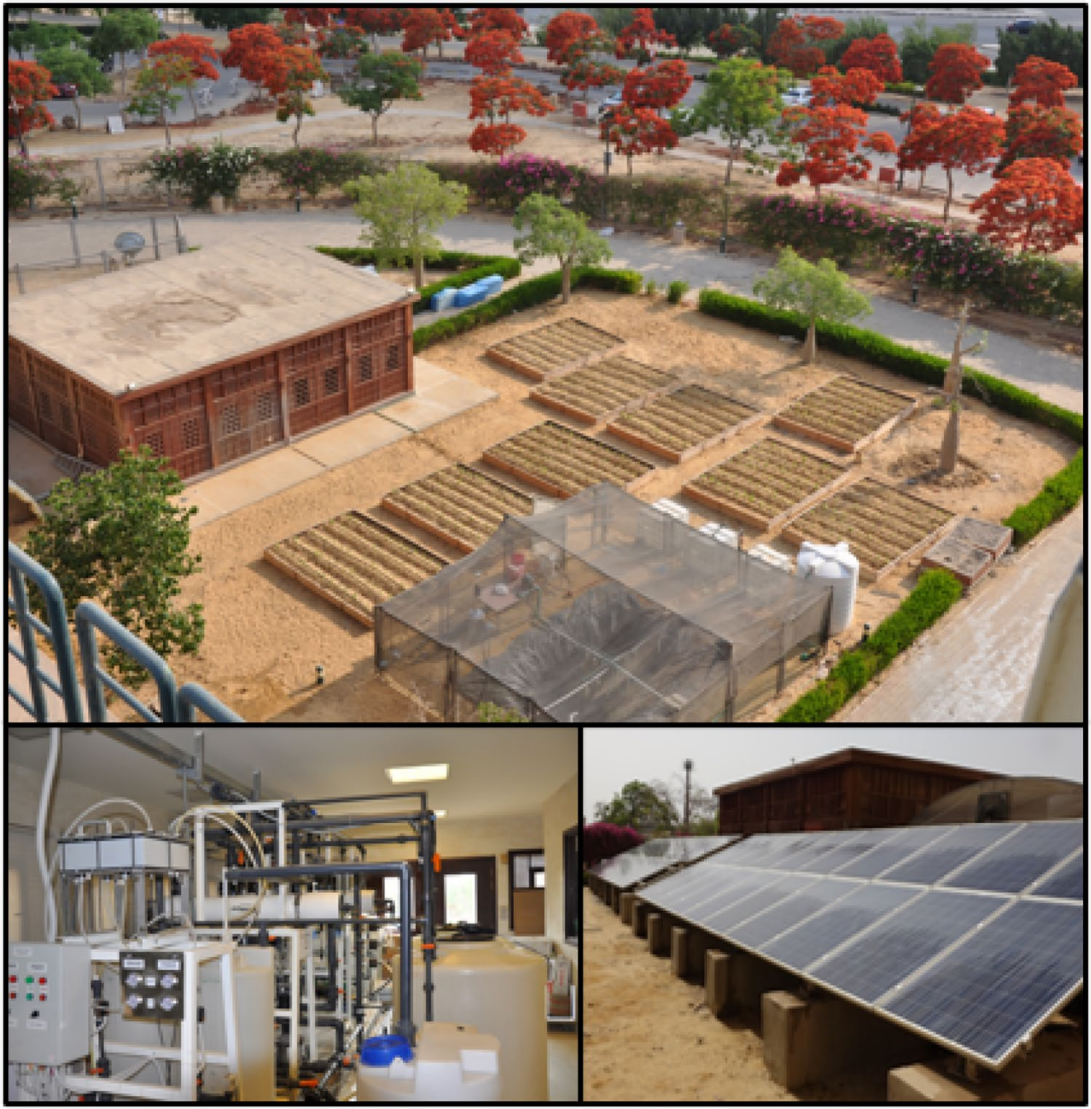
The proposed Water, Energy and Food Nexus model

This study aims to investigate the productivity of the integrated solar-powered water desalination, aquaculture, and agriculture under the Water-Energy-Food Nexus’ umbrella. This work’s long-term goal is to establish an integrated approach to resolve water, energy, and food challenges and consider their interconnectedness to develop a sustainable crop production model (Fig. 1).

## Materials and methods

### Study location

The field experiment was conducted at the Research Institute of the Center for Applied Research on the Environment and Sustainability at The American University in Cairo, New Cairo, Egypt (30°01′11.7″N 31°29′59.8″E), in the growing season of Spring 2019.

### Model layout

**Fig. 2:**
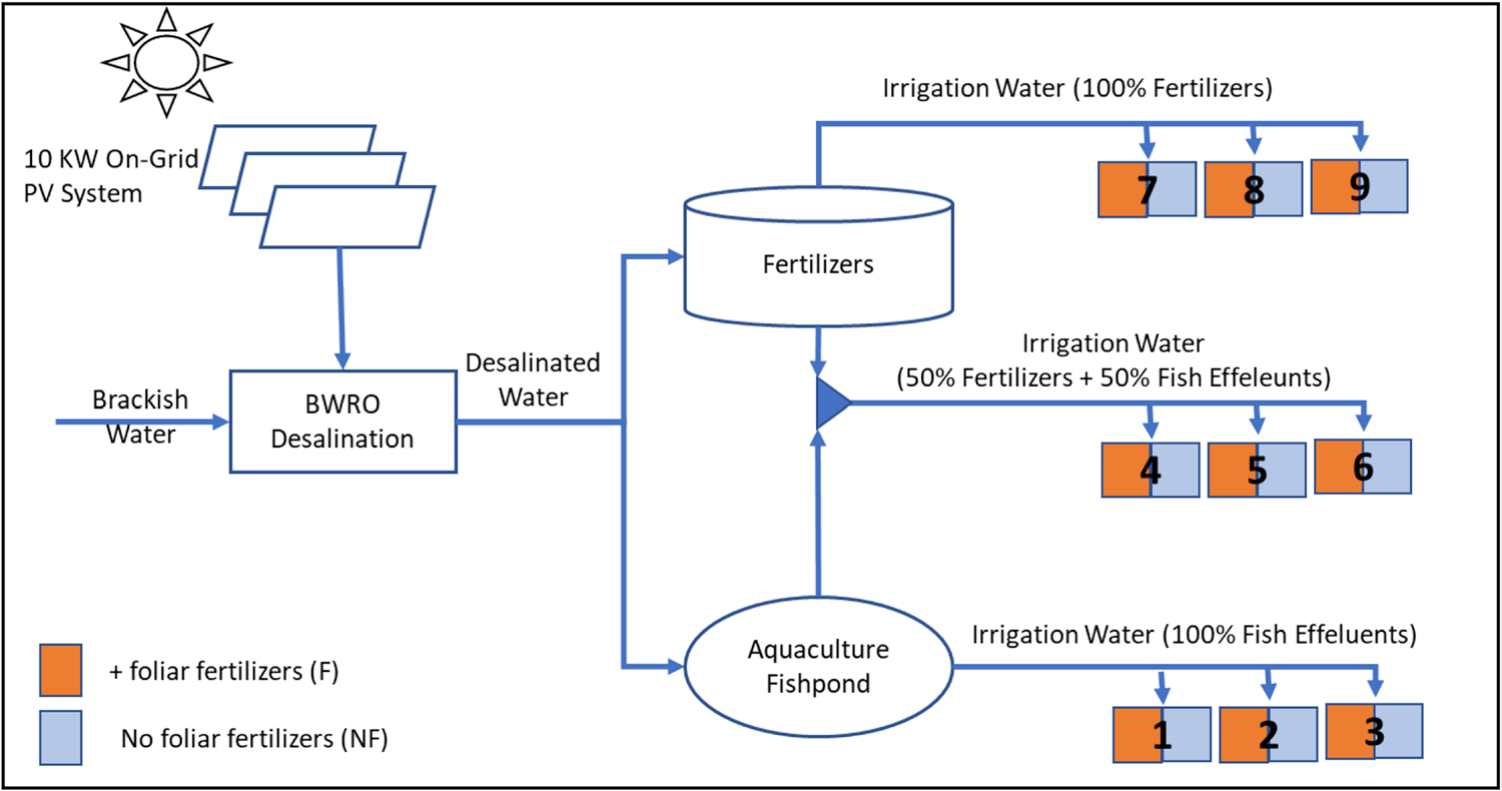
Schematic integrated model design

In Fig. 2, the proposed design starts by treating brackish water using RO membrane separation technology, powered by an on-grid 10-kW photovoltaic solar panel. The permeate (freshwater) from the RO facility is divided into two parts. The first part is directed to an aquaculture fishpond, where the fish effluents are used as irrigation water for the crop beds. The second part of the permeate is used for fertigation with inorganic fertilizers. Three different treatments have been conducted in this system: (a) fish effluents as 100% source of fertilizers (ETs), (b) a mixed treatment (MT) of both effluents and chemical fertilizers at 50% application each, (c) irrigation using 100% standard recommended chemical fertilizers (CTs). The effluents for both ET and MT were sourced from the fishpond and compensated back to the fishpond by new freshwater (permeate). The experiment was conducted following a split-plot design with three factors, i.e., three main plots (fertilizer treatments; ET, MT, and CT) and two sub-plots (with foliar nutrient (F) application or without (NF)) under three replications.

Each main plot was subdivided into two sub-plots; one sub-plot was applied with foliar fertilizers (magnesium (Mg), calcium (Ca), zinc (Zn), manganese (Mn), and iron (Fe)), and the second sub-plot without foliar application. The total number of plots were nine, i.e., the three main plots under three replications and their subsequent sub-plots (effluent treatment with foliar application (ET-F), effluent treatment without foliar application (ET-NF), control treatment with foliar application (CT-F), control treatment without foliar application (CT-NF), mixed treatment with foliar application (MT-F), mixed treatment without foliar application (MT-NF)).

The chemical fertilizers were added according to Egypt’s recommended rate (Kintzios 2003; FAO 2005). Compost was also added at a rate of 85 m^3^/ha 2 weeks prior transplanting for every experimental bed. The chemical analysis of experimental soil for the three treatments was as follows: pH 7.93, EC 6.22; anions (meq./L)—HCO_3_^−^ 4.32, SO_4_^2−^ 34.32, Cl^−^ 36; cations (meq./L)—Ca^2+^ 19.5, Mg^2+^ 13.01, Na^+^ 40.5, N 81.0, P 8.18, and K 112.6 meq./L.

### Desalination

The desalination process is indirectly powered using a grid-tied solar photovoltaic system consisting of 41 poly-crystalline Suntech modules (Model STP-255), each with a dimension of 1.64 × 0.99 m and a rated power equivalent to 255 W. The modules are connected to a 10-kW Fronius Symo on-grid inverter, converting DC to AC. The produced electricity is fed into the grid to offset the consumption of all the electrical loads within the experiment site.

The desalination process was conducted by RO in batch mode using a Sea Water Pump with Energy Recovery Unit (model Danfoss-APP1.0/APM1.2). The RO membrane used is Hydraunatic SWC5-4040, three modules were connected in a series arrangement (3 pressure vessels each equipped with a single module). Synthesized brackish water was prepared by dissolving industrial-grade sodium chloride (sea salt) from El-Arish Governorate, Egypt. The salt chemical properties are presented in Table 1. Feedwater salinity was 10 g/L (typical brackish water in the region), with an equivalent osmotic pressure equal to 8.61 bars. The osmotic pressure was calculated using Van’t Hoff equation. Permeate TDS was 103 mg/l, and brine TDS was 12.4 g/l. All experiment runs were performed at 25 °C.

**Table 1:** Chemical properties of the used salt

Elements in the dry salt sample			
Sodium chloride	98.5%	Bicarbonate	0.004%
Moisture	0.23%	Iron	0.03 mg/kg
Insoluble matter	0.02%	Copper	0.02 mg/kg
Soluble matters	0.57%	Arsenate	0.2 mg/kg
Calcium	0.0721%	Lead	0.2 mg/kg
Magnesium	0.0722%	Mercury	0.05 mg/kg
Sulfate	0.313%	KIO_3_	53 ppm
Potassium	0.02%	Cadmium	0.008 mg/kg
Carbonate	-		

### Aquaculture pond

Produced freshwater from the RO facility is directed to an aquaculture tilapia fishpond. A trapezoidal-lined pond was used with dimensions 3 m by 2 m and 1.5-m depth to cultivate Nile tilapia (*Oreochromis niloticus*) at a stocking density of 60 fish/m^3^ at the fingerlings stage on 19 May 2019. The pond was lined with high-density polyethylene plastic with 1-mm thickness to avoid seepage and is equipped with a submerged water pump to lift water through the water filter to the drip irrigation system, eventually irrigating the crops. The pond was also equipped with two sedimentation tanks and a mechanical filter for continuous water recycling. The fish were fed 3–4 times daily with commercial pellets containing 30% proteins, 5% crude lipid, 6% crude fiber, 13% ash, and 9% moisture content supplied by Skretting Egypt. The feeding pattern and frequency was according to the fish body biomass percentage of 2–4% depending on the growth stage and upon reaching satiation.

Water quality parameters in the fishpond such as water temperature, pH, and dissolved oxygen (DO) were closely monitored using automated digital Nilebot technologies by Conative labs. In contrast, ammonia, nitrite, and nitrate were monitored using an API test kit every week. These parameters’ recorded values were as follows: water temperature ranged between 23 and 28 °C, DO range between 6 and7 mg/L, and pH between 6.5 and 7.5. Ammonia and nitrite levels were kept below 1 mg/L. Table 2 shows the chemical water properties of the effluents from the fishpond.

**Table 2:** Chemical properties of water samples used

Element	Feedwater	Brine	Permeate (influent into fishpond)	Effluent from the fishpond		
				Start experiment	Mid experiment	End experiment
EC (dS/m)	13.9	16.4	0.3	0.55	0.67	0.57
pH	7.71	7.51	8.12	7.23	7	6.77
ppm	10,120.0	13,120.0	192.0	352	429	365
HCO_3_^−^ (meq/L)	1.89	3.78	1.31	2.16	2.16	1.89
Cl^−^ (meq/L)	130	125	1.5	2	2	2
SO_4_^−^ (meq/L)	76.6	117.22	0.2	1.34	2.54	1.81
Ca^++^ (meq/L)	12.7	7.57	0.4	2.38	2.4	2.65
Mg^++^ (meq/L)	10.62	8.28	0.26	1,51	2.59	1.24
Na^+^ (meq/L)	1.85	230	2.34	1.3	1.4	1.5
K^+^ (meq/L)	0.16	0.15	–	0.31	0.31	0.31
NH4^+^ (mg/L)	˂ 0.1	1.26	0.42	˂ 0.1	2.8	˂ 0.1
NO_3_^−^ (mg/L)	3.22	6.44	8.96	5.67	10.5	4.9
P (mg/L)	˂ 1.5	˂ 1.5	˂ 1.5	˂ 1.5	˂ 1.5	˂ 1.5
Fe (mg/L)	0.145	0.149	0.136	0.162	0.274	0.145
Mn (mg/L)	˂ 0.5	˂ 0.5	˂ 0.5	˂ 0.5	˂ 0.5	˂ 0.5
Zn (mg/L)	˂ 0.2	˂ 0.2	˂ 0.2	˂ 0.2	˂ 0.2	˂ 0.2
Cu (mg/L)	˂ 0.2	˂ 0.2	˂ 0.2	˂ 0.2	˂ 0.2	˂ 0.2
B (mg/L)	˂ 0.3	˂ 0.3	˂ 0.3	˂ 0.3	˂ 0.3	˂ 0.3
SAR	54.3	81.56	4.11	0.93	0.89	1.07

#### Measurements for fish growth performances

Survival, mortality rates, and specific growth rates.

After the fish growing period of 182 days, fish growth–related parameters were calculated and recorded using the following equations presented by Liu et al. (2017):

$$\frac{\mathrm{i}.\mathrm{\;Survival\;Rate\;}(\mathrm{\%}) =\mathrm{ Number\;of\;Fish\;at\;the\;End\;of\;the\;Experiment}}{\mathrm{Number\;of\;Fish\;at\;the\;beginning\;of\;the\;Experiment}} \times 100$$

$$\frac{\mathrm{ii}.\mathrm{\;Mortality\;rate\;}(\mathrm{\%}) =\mathrm{ Number\;of\;Fish\;that\;died\;during\;the\;experiment}}{\mathrm{Number\;of\;Fish\;at\;the\;beginning\;of\;the\;Experiment}} \times 100$$

$$\frac{\mathrm{iii}.\mathrm{\;Specific\;Growth\;Rate }\;(\mathrm{SGR}) =\mathrm{ ln\;}(\mathrm{Final\;Mean\;Weight}) -\mathrm{ ln\;}(\mathrm{Initial\;Mean\;Weight})}{\mathrm{Length\;of\;Feeding\;Trial\;}(\mathrm{days})}$$

### Plants

Basil seedlings aged 60 days of uniform size and heights were collected from the Horticultural Institute at the Agricultural Research Center in Dokki, Egypt. These were uniformly selected and transplanted into isolated experimental plots of 8m^2^. The seedlings were transplanted at 60 cm between rows and 30 cm between hills on 26 May 2019. Thinning to one plant per hill was performed at 40 days after transplanting when the plants had been firmly established. Data for relative plant growth parameters were recorded at different stages throughout the whole growth cycle. Parameters included plant height, number of branches, and chlorophyll content. Two harvests or cuts were carried out whenever the plants reached the harvesting stage at 70% flowering. At every harvest, plants were cut 5 cm above the soil surface, fresh and dry weights of plants were measured and recorded (g plant^−1^ and t ha^−1^), and the number of leaves per plant recorded.

### Chemical analysis

#### Water and soil analysis

Standard methods and portable meters were used to measure the different chemical compositions of the water samples. Chlorophyll concentration was measured in SPAD units using MC-100 Chlorophyll meter from Apogee Instruments, Inc. by taking average measurements for three well-developed leaves.

Soil samples were analyzed at the Agricultural Research Center, Giza, Egypt. The soil particle size distribution was carried out using the pipette method. Electrical conductivity values were measured from the soil paste extract; soil pH values were taken from soil suspensions at ratio 1:2.5 as described by Estefan (2013). The available nitrogen in the soil sample was extracted using potassium chloride (KCl) as an extractable solution with the ratio of (5 g soil to 50 ml KCl) and determined using the micro-Kjeldahl method. Available potassium was determined using a flame photometer, and the other elements in the soil sample were determined by using inductively coupled plasma spectrometry (model Ultima 2 JY Plasma) (EPA 1991; Soltanpour 1991).

### Essential oil extraction and gas chromatography–mass spectrometry analysis

Essential oils were extracted from samples of 100 g fresh weight (a combination of stems, leaves, and inflorescences) in triplicates for each sub-treatment/sub-plot and expressed as a percentage per 100 g sample. They were hydro-distilled over 90 min using 500 mL of distilled water with a fixed extraction time of 3 h. The distillate was extracted with diethyl ether and dried over anhydrous sodium sulfate. The resulting organic layer of essential oils was concentrated at 30 °C using a vigreux column. The oil was then weighed, and the essential oil content was expressed in % (volume/weight). The gas chromatography–mass spectrometry analysis (GC–MS) analysis was performed using Agilent Technologies equipped with gas chromatography (7890B) and mass spectrometer detector (5977A) at Central Laboratories Network, National Research Centre, Cairo, Egypt. Analysis was performed as described in Adams (2005) and Kimera et al. (2021b).

### Statistical analysis

Data analysis followed a completely randomized design framework with two factors, i.e., treatments (ET-F, ET-NF, CT-F, CT-NF, MT-F, and MT-NF) and foliar nutrient application with three replications. Analysis of variance and *T*-tests were performed using SPSS V22. All the extractions were analyzed in triplicates using data expressed as mean ± S.D with Duncan test at α = 0.05.

## Results and discussion

### Desalination and fish rearing unit

The fact that desalination was performed in batches, each run produced around 4 m^3^ of permeate, which was enough to irrigate the designated plant area. The recorded average permeate recovery for the RO process is 22%, and salt rejection exceeded 98.7%. The differential pressure between membrane inlet and outlet was equal to 1 bar, where membrane inlet pressure was 16 bars, and the outlet was 15 bars. The average transmembrane pressure was equal to 16 bars and an average permeate and brine flow rates equivalent was 3.49 and 12.41 LPM, respectively. The average pure water flux was 9.5 LMH. The recorded permeate salinity was 200 ppm.

Tilapia was stocked in 6m^3^ water volume in a fishpond (60 fish m^−3^) and cultured for 6 months. At harvesting, the average fish harvest mass was 0.458 kg, with a survival rate of 97.7% and specific growth rate of 1.1% at *N* = 30. The few recorded mortality was in the first week of culturing during the acclimatization phase. The water quality was sufficient for the fish’s growth, and more so, the produced fish was of normal and healthy status. At the end of the experiment, the total harvest yield of fish was 150 kg representing 25 kg/m^3^ of water.

### Plant growth and yield

Plants require specific nutrients to grow and establish; some of these nutrients are needed in high quantities such as nitrogen, potassium, and phosphorous whereas others are needed in low quantities such as iron, manganese, and zinc. Some of these elements can be supplied from the fish effluents while some can be supplemented. Like most other fish species, tilapia only utilizes 20–30% of the nitrogen supplied from the fish feed. An approximate 70–80% of the nitrogen is released in wastewater in the form of ammonia that is converted into nitrates facilitated using biofilters for systems such as aquaponics or biofloc systems (Krom et al. 1995; Piedrahita 2003; Schneider et al. 2005; Ngo Thuy Diem et al. 2017). However, in IAA, the whole nitrogen reaction chain naturally occurs in the soil system (Prein 2002; Dey et al. 2010; Ahmed et al. 2014b). It is important also to note that many studies have reported the deficiency of some minerals in crops solely irrigated with fish wastewater, including calcium, magnesium, potassium, manganese, and iron (Seawright et al. 1998; Graber and Junge 2009; Kasozi et al. 2019; Kaburagi et al. 2020). Deficiency of some elements such as K, Mn, S, and Fe in fish effluents is a negative reality, most especially if dealing with fruiting vegetables; its deficiency symptoms are easily recognized in fruits and plant leaves (Rakocy et al. 2006; Nelson and Pade 2008; Roosta and Hamidpour 2011).

### Number of branches and plant height

In comparison among the three main treatments, ET exhibited the highest branching and plant height irrespective of the cut number, reaching 17.7 branches and 62-cm height, followed by MT and CT. Generally, all treatments gave the highest values when applied with foliar fertilizers. For instance, in cut 2, ET-F recorded the highest number of branches, i.e., 17.7 branches, followed by ET-NF (16 branches), MT-F (15.7 branches), CT-F (13.6 branches), MT-NF (12.5 branches), and finally CT-NF (11.8 branches). Furthermore, the sub-treatments “with foliar” showed a higher number of branches than the “without foliar” sub-treatment.

**Fig. 3:**
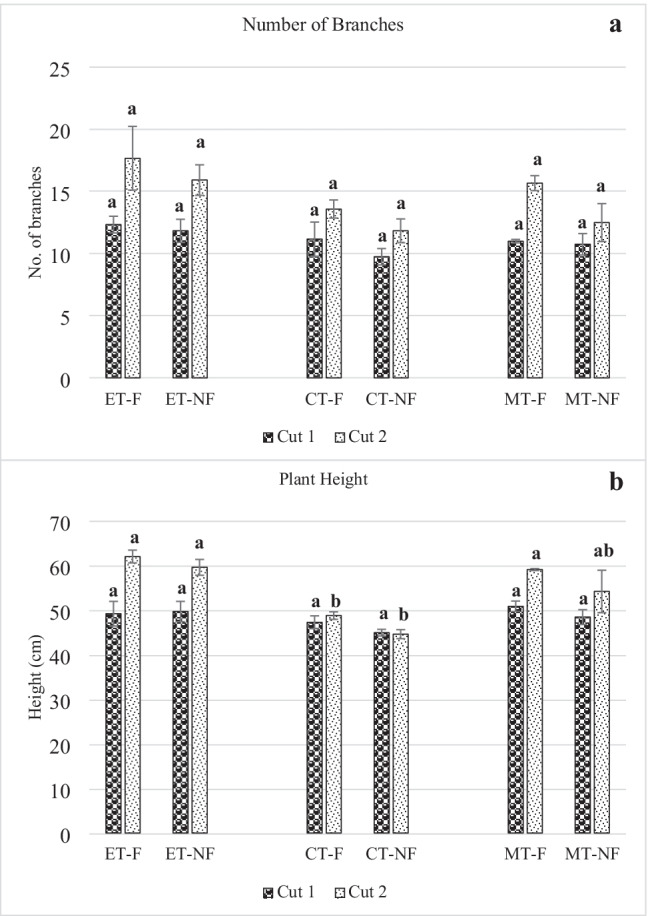
Number of branches and crop height for the three treatments in the two cuts

As for plant height, ET-F in cut 2 exhibited the highest plant height exceeding 62 cm, closely followed by ET-NF and MT-F at 60 cm and 59 cm, respectively, as shown in Fig. 3. However, cut 2 showed higher plant heights than cut 1. For example, CT-F at the early crop stage was 14.6 cm in cut 1, while it was 24.5 cm in cut 2. Concerning the type of treatment, both effluent and mixed treatment exhibited close results, and chemical treatment had the least plan height. Foliar application has a minor effect on plant height; for example, ET-F and ET-NF in cut 1 have almost similar results. Saha et al. (2016) reported that their basil plants reached 89.9 and 78.7 cm in aquaponics and hydroponics experiments, respectively. This is contrary to our findings, as the maximum height attained was 62 cm/plant. However, similar to Sharafzadeh and Omid (2011) and El-Naggar et al. (2015), they reported that sweet basil could grow to 50–60 cm in height with optimum growing conditions.

#### Fresh and dry crop yield

**Fig. 4:**
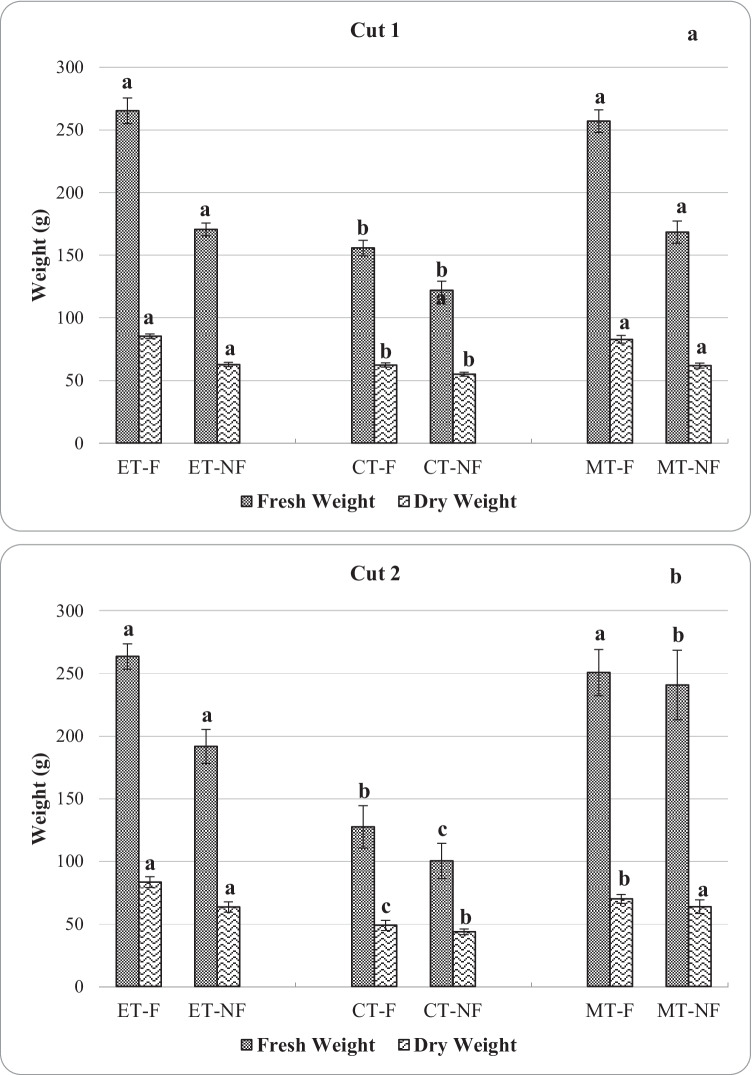
The fresh and dry weight of basil for both cuts

The effluent treatment under foliar application recorded the highest fresh and dry weights of basil, followed by the mixed treatment, and then the chemical treatment as illustrated in Fig. 4. For instance, in cut 1, CT-F recorded significantly lower yields 156 g/plant, and 62 g/plant (8.65 ton/ha and 3.45 ton/ha) compared to ET-F with 266 g/plant, and 85 g/plant (14.73 ton/ha and 4.74 ton/ha) and MT-F at 257 g/plant, and 83 g/plant (14.27 ton/ha and 4.6 ton/ha) for fresh and dry weights, respectively. However, there was no significant difference observed between the fresh and dry weights of ET-F and MT-F in cut 1. Similar statistical results were observed for the non-foliar treatments between ET-NF, CT-NF, and MT-NF in cut 1. The average fresh weights from ET-NF were 171 g/plant (9.47 ton/ha), CT-NF was 122 g/plant (6.77 ton/ha), and MT-NF recorded 168 g/plant (3.44 ton/ha). Comparing foliar and non-foliar sub-treatments for the same treatments shows that most of the treatments had significant results. This is true for fresh weights of ET-F and ET-NF, CT-F and CT-NF, MT-F, and MT-NF. They were all significantly different at *p* < 0.05. The same analysis trend was true for the dry weights.

Regarding the second cut, ET-F significantly produced the highest fresh weights with 263 g/plant (14.63 ton/ha), compared to CT-F, which yielded 127 g/plant (7.07 ton/ha). This is more than a 100% difference in yield, although there was no significant difference between ET-F and MT-F at *p* > 0.05. Contrary, all treatments (ET-F, MT-F, and CT-F) had significant differences in their dry weights as shown clearly in Fig. 4. There was a significant difference between all treatments with the non-foliar sub-treatments. ET-NF produced almost double the yield of CT-NF. ET-NF was significantly higher in fresh weights than MT-NF and CT-NF. CT-NF exhibited the lowest fresh and dry weights equivalent to 100 g/plant and 44 g/plant, respectively, in cut 2. Furthermore, comparing whether the foliar application and no foliar application had significant differences between treatments, it was found that ET-F and ET-NF had significant differences between their fresh weights. In contrast, CT-F and CT-NF, in addition to MT-F and MT-NF, had no significant difference at *p* > 0.05. There was a significant interaction between treatments and both the fresh and dry weights (*p* < 0.0001).

The foliar application significantly affects both fresh and dry weights in cut 2 under CT and MT. Yet, foliar application in cut 1 did not significantly impact fresh and dry weights under all treatments. Several authors have reported the significant increase in fresh biomass of crops due to foliar fertilizers on crops such as basil, tomatoes, maize, and wheat (Kaya et al. 2001a, b; Ling and Silberbush 2002; Mosali et al. 2006; Fageria et al. 2009). This study findings show that basil can reach over 263 g/plant (14.63 ton/ha) of fresh weight in the first cut using effluents. These results are far greater than those reported by Saha et al. (2016) in their greenhouse experiment regarding basil plants. They also recorded the highest fresh yield of basil attained in aquaponics at 8.34 ton/ha and 5.36 ton/ha in hydroponics. This shows that the current study findings produced fresh basil biomass of 64% and 73% more than the earlier reported yield in aquaponics and hydroponics. They have demonstrated that basil cultivated under an aquaponic system resulted in significantly the highest plant heights (14%), fresh weight (56%), and dry weight (65%) compared to the hydroponic system except for leaf nutrients and chlorophyll content. In another experiment, Ekren et al. (2012) only reported 0.4 ton/ha and 0.3 ton/ha of fresh and dry basil respectively when investigating the irrigation deficiency in purple basil. On contrary, Sabry et al. (2019) reported very high yields up to 27.25 ton/ha and 5.9 ton/ha for fresh and dry yields with sweet genovese basil variety. Even though almost all famers would regard fish effluents as wastes, this study demonstrated that 1 m^3^ of fish effluents can yield 5 kg of basil with supplemental foliar applications or 3.5 kg of fresh basil without any additional foliar fertilizers. These figures can still increase in case of more than two cuts per season.

### Number of leaves/plants

**Fig. 5:**
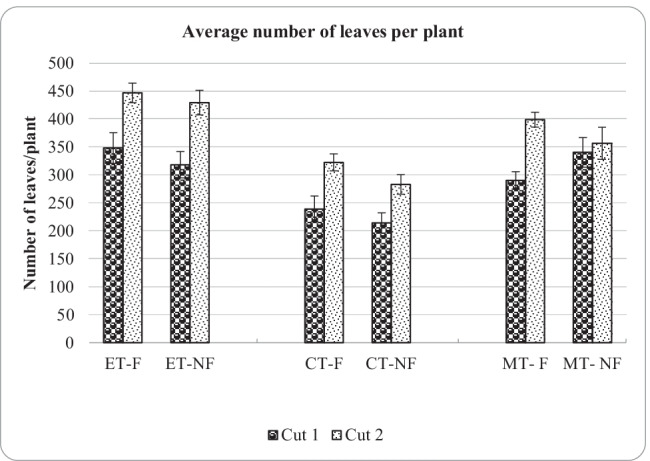
Number of leaves at the Harvesting time for all the treatments for two cuts

Additionally, the number of leaves per plant for all the treatments was counted at harvesting time, and the average figures are illustrated in Fig. 5. Since some crop applications are based on using fresh or dry leaves, it was also necessary to report the yield in terms of leaves harvested.

ET exhibited the highest number of leaves of around 447 leaves/plant under foliar application, followed by MT and lastly CT. There was a significant difference in the number of leaves between the different treatments. The foliar application proved to have a positive effect on leafing factor of the crop within other treatments. For example, leafing of CT in cut 2 increased from 239 to 323 leaves/plant (68% increase) due to foliar application. An exception to this trend was MT in cut 1, in that MT-NF recorded a higher number of leaves than MT-F. On the other hand, ET-F recorded a 64% increase in the number of leaves from cut 1 to cut 2, while ET-NF recorded a 68% increment. There was a significant interaction between the number of leaves and the cut number.

### Chlorophyll content in leaves

Chlorophyll is an essential photosynthetic pigment in plants. The amount of chlorophyll inside the plant leaves also indicates how healthy and nourishing the plant is. Table 3 summarizes the chlorophyll measured in plants from the different treatments.

**Table 3:** Chlorophyll content in SPAD units

Treatments	Foliar	Non-foliar
CUT 1		
ET	318.54^a^ ± 18.02	282.33^a^ ± 30.64
CT	231.06^a^ ± 12.21	244.58^a^ ± 12.53
MT	282.36^a^ ± 19.51	268.82^a^ ± 15.07
CUT 2		
ET	326.03^a^ ± 49.33	309.68^a^ ± 7.55
CT	238.47^a^ ± 10.89	208.20^b^ ± 17.60
MT	311.40^a^ ± 6.09	296.78^a^ ± 9.07

Data is expressed as means ± standard error (SE). Different lower superscript letters within each treatment accross the rows indicate a significant difference at *P* < 0.05 for each parameter (Duncan Multiple range test)

Results showed no significant differences between chlorophyll content treatments except in cut 2 where CT-NF resulted in a significantly lower amount in chlorophyll content. Although analytical results did not show much significance in chlorophyll content, it was physically observed that the ET treatment crops were greener in color than those in MT and CT. Some researchers have demonstrated an effect of micro-element spray on leaf chlorophyll. Roosta and Hamidpour (2011) reported that foliar application of Mn or Fe has a significant effect on the content of chlorophyll in tomato plants grown under hydroponics.

### The essential oil content in plant sample

**Fig. 6:**
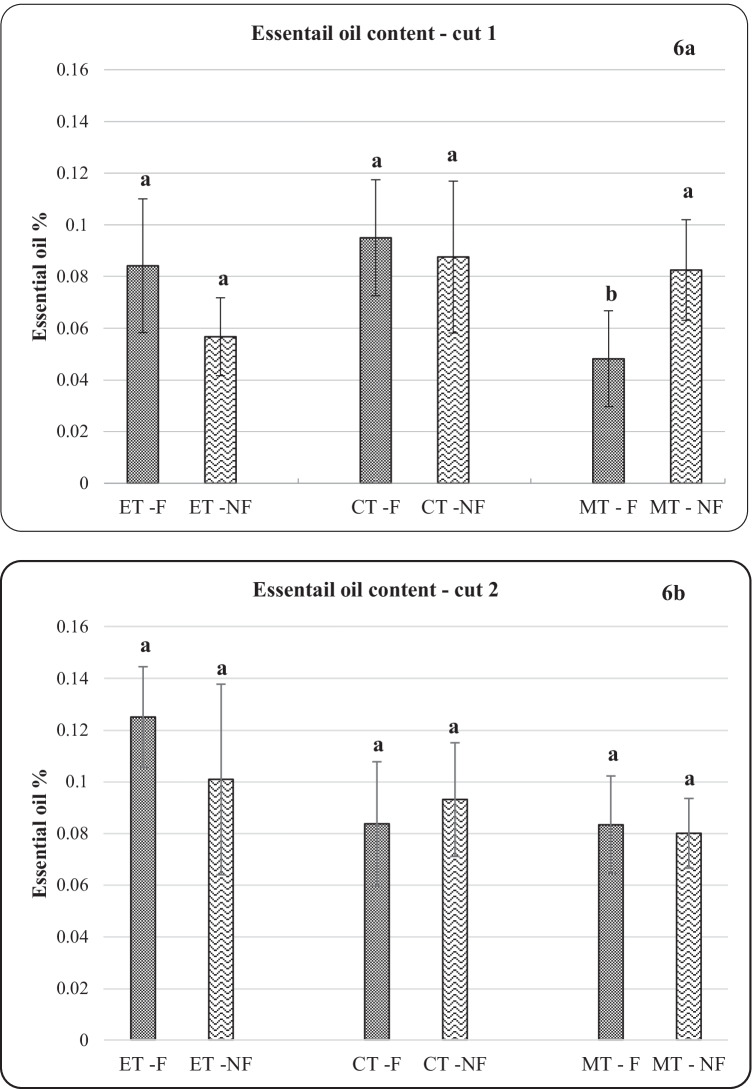
Essential oil (%) for the two cuts for the different treatments

The economic importance of basil is attributed to its EO content, which varies depending on the genotype, growth media, and climatic conditions. In the first cut, irrespective of the sub-treatment, CT resulted in the highest percentage of essential oils extracted from 100 g of plant samples. CT-F yielded 0.095% compared to 0.084% and 0.049% for ET-F and MT-F respectively. These values are far less than what was reported in by Gürbüz et al. (2009b). However, frequent use and unbalanced application of these inorganic fertilizers, mostly in intensive farming systems, coupled with inadequate and poor farming practices, can result in unsustainable production in addition to environmental and soil pollution (Akram Qazi et al. 2009; Malik et al. 2011). Even though the numbers in Fig. 6 show numerical variations, SPSS analysis showed no significant differences in essential oils between treatments concerning either foliar or non-foliar applications, except for only MT treatment. Results show that MT-NF was significantly higher in oil yield compared to MT-F in cut 2.

### Essential oil composition

Basil essential oils are used in several applications, from perfumery, medicinal, cosmetics, and food preservation. Results in Table 4 show the different composition elements in the oil samples analyzed from all the treatments in both cuts. Irrespective of the treatments, the most dominant elements from this study were linalool (55.5–61.6%), tau.-cadinol (5.3–8.3%), eucalyptol (5.4–7.7%), eugenol (2–6.8%), (Z,E)-.alpha.-farnesene (3–5.2%), and beta.-elemene (2.4–3.5%). Almost the most significant percentage of these major elements/constituents was observed from the ET and MT treatments. Linalool was highest in ET-F at 61.6%, followed by tau-cardinol with its highest composition at 8.8% in MT-NF, eucalyptol at 6.9% ET-NF, and then eugenol with 6.8% under the ET-NT treatment. The six main constituents were analyzed statistically, and the results showed that they had no significant differences between the treatments.

**Table 4:** The chemical composition of the essential oil from different treatments for cut 1 (a) and cut 2 (b)

Compound	ET-F	ET-NF	CT-F	CT-NF	MT-F	MT-NF
(a) Essential oil composition cut 1						
.Alpha.-pinene, (-)-	0.239 ± 0.058	0.245 ± 0.096	0.322 ± 0.119	0.350 ± 0.119	0.238 ± 0.111	0.178 ± 0.141
Camphene	0.057 ± 0.021	0.050 ± 0.016	0.066 ± 0.032	0.085 ± 0.025	0.046 ± 0.023	0.078 ± 0.013
Sabinene	0.194 ± 0.089	0.193 ± 0.05	0.690 ± 0.309	0.378 ± 0.272	0.291 ± 0.305	0.293 ± 0.267
.beta.-Pinene	0.535 ± 0.138	0.612 ± 0.181	0.755 ± 0.049	0.670 ± 0.192	0.542 ± 0.123	0.485 ± 0.173
.beta.-Myrcene	0.158 ± 0.056	0.186 ± 0.091	0.318 ± 0.079	0.349 ± 0.131	0.190 ± 0.117	0.223 ± 0.129
d-Limonene	0.200 ± 0.049	0.260 ± 0.076	0.8727 ± 0.701	0.696 ± 2.345	0.160 ± 0.014	0.230 ± 0.043
Eucalyptol	6.377 ± 2.36	6.966 ± 2.388	5.458 ± 2.856	7.728 ± 2.966	6.625 ± 1.239	5.701 ± 2.332
.beta.-Ocimene	1.188 ± 0.406	1.418 ± 0.374	1.281 ± 0.496	1.478 ± 0.637	1.449 ± 0.411	1.166 ± 0.652
Cyclohexene, 3-methyl-6-(1-methylethylidene)-	0.099 ± 0.023	0.097 ± 0.031	5.724 ± 1.934	5.280 ± 1.779	0.118 ± 0.029	0.103 ± 0.052
Linalool	61.606 ± 9.199	61.175 ± 7.356	56.60 ± 18.278	55.993 ± 18.566	55.52 ± 5.017	58.041 ± 15.68
Myroxide	0.256 ± 0.079	0.170 ± 0.092	0.352 ± 0.125	0.480 ± 0.388	0.288 ± 0.096	0.277 ± 0.089
( +)-2-Bornanone	1.287 ± 0.177	1.133 ± 0.225	1.194 ± 0.413	1.228 ± 0.450	1.368 ± 0.204	1.178 ± 0.465
1,4-Pentadiene, 2,3,3-trimethyl-	0.029 ± 0.008	0.036 ± 0.015	0.123 ± 0.059	0.130 ± 0.066	0.048 ± 0.024	0.086 ± 0.05
Terpinen-4-ol	0.270 ± 0.354	0.048 ± 0.05	0.212 ± 0.353	0.152 ± 0.236	0.197 ± 0.413	0.186 ± 0.295
Terpineol	0.589 ± 0.123	0.626 ± 0.227	0.767 ± 0.156	0.774 ± 0.181	0.707 ± 0.15	0.829 ± 0.172
Bornyl acetate	0.897 ± 0.238	0.546 ± 0.284	0.858 ± 0.196	0.683 ± 0.193	0.879 ± 0.258	0.718 ± 0.266
1,5-Heptadiene, 2,5-dimethyl-3-methylene-	0.644 ± 0.247	0.662 ± 0.277	0.768 ± 0.362	1.040 ± 1.117	0.860 ± 0.407	0.878 ± 0.958
Eugenol	4.690 ± 4.383	6.890 ± 4.523	2.992 ± 3.299	1.995 ± 1.628	6.332 ± 0.525	1.984 ± 1.665
Phenol, 2-methoxy-4-(1-propenyl)-	0.101 ± 0.078	0.087 ± 0.101	0.075 ± 0.061	0.101 ± 0.66	0.094 ± 0.066	0.088 ± 0.049
1,11-Dodecadiyne	0.207 ± 0.087	0.206 ± 0.092	0.471 ± 0.768	0.379 ± 0.435	0.581 ± 0.743	0.335 ± 0.330
.beta.-Elemene	2.700 ± 0.883	2.592 ± 0.991	2.474 ± 1.042	2.399 ± 0.969	2.780 ± 1.399	3.502 ± 3.291
.beta.-Ylangene	0.422 ± 0.123	0.404 ± 0.155	0.502 ± 0.175	0.958 ± 1.827	0.466 ± 0.184	0.594 ± 0.459
.beta.-CUBEBENE	0.155 ± 0.049	0.457 ± 0.873	0.230 ± 0.046	0.191 ± 0.065	1.512 ± 2.92	0.257 ± 0.197
(Z,E)-.alpha.-farnesene	4.2564 ± 2.675	3.061 ± 2.554	3.576 ± 3.284	4.033 ± 3.123	3.785 ± 3.312	5.251 ± 2.796
Aromandendrene	0.410 ± 0.129	0.421 ± 0.172	0.408 ± 0.166	0.412 ± 0.171	0.494 ± 0.195	0.568 ± 0.616
cis-Muurola-3,5-diene	0.160 ± 0.064	0.152 ± 0.049	0.194 ± 0.200	0.178 ± 0.139	0.249 ± 0.167	0.202 ± 0.152
Humulene	0.825 ± 0.263	0.754 ± 0.295	0.760 ± 0.276	0.715 ± 0.257	0.907 ± 0.283	1.097 ± 0.862
Bicyclosesquiphellandrene	0.298 ± 0.107	0.299 ± 0.086	0.390 ± 0.339	0.409 ± 0.328	0.481 ± 0.367	0.442 ± 0.309
Germacrene D	1.500 ± 0.443	1.379 ± 0.491	1.475 ± 0.460	1.460 ± 0.523	1.740 ± 0.504	2.045 ± 1.663
gamma.-Elemene	0.578 ± 0.195	0.604 ± 0.239	0.587 ± 0.207	0.558 ± 0.209	0.731 ± 0.205	0.822 ± 0.899
alpha.-Bulnesene	0.670 ± 0.243	0.674 ± 0.278	0.802 ± 0.281	0.798 ± 0.295	0.952 ± 0.32	1.022 ± 0.989
gamma.-Muurolene	1.448 ± 0.459	1.448 ± 0.521	1.454 ± 0.569	1.556 ± 0.518	1.672 ± 0.509	2.305 ± 1.88
cis-Calamenene	0.235 ± 0.135	0.243 ± 0.129	0.236 ± 0.124	0.312 ± 0.106	0.289 ± 0.112	0.417 ± 0.173
1H-3a,7-Methanoazulene, octahydro-1,4,9,9-tetramethyl-	0.398 ± 0.308	0.190 ± 0.179	0.278 ± 0.147	0.291 ± 0.146	0.396 ± 0.189	0.379 ± 0.246
Cubenol	0.500 ± 0.253	0.988 ± 1.619	0.879 ± 1.063	0.923 ± 1.243	1.037 ± 1.485	0.742 ± 0.426
.tau.-Cadinol	6.108 ± 2.903	5.366 ± 3.288	5.693 ± 2.533	5.252 ± 2.01	6.221 ± 2.658	8.302 ± 4.895
.alpha.-Cadinol	0.126 ± 0.109	0.174 ± 0.148	0.233 ± 0.080	0.219 ± 0.064	0.174 ± 0.107	0.262 ± 0.228
1,9-Decadiyne	0.163 ± 0.245	0.036 ± 0.018	0.146 ± 0.128	0.075 ± 0.035	0.042 ± 0.017	0.050 ± 0.029
(b) Essential oil composition cut 2						
.Alpha-pinene, (-)-	0.469 ± 0.242	0.425 ± 0.142	0.301 ± 0.115	0.279 ± 0.135	0.313 ± 0.124	0.324 ± 0.082
Camphene	0.206 ± 0.061	0.153 ± 0.054	0.130 ± 0.052	0.108 ± 0.056	0.110 ± 0.048	0.128 ± 0.042
Sabinene	0.222 ± 0.121	0.338 ± 0.136	0.241 ± 0.103	0.203 ± 0.106	0.235 ± 0.124	0.228 ± 0.086
.beta.-Pinene	1.063 ± 0.412	0.818 ± 0.277	0.558 ± 0.209	0.516 ± 0.223	0.591 ± 0.240	0.585 ± 0.171
.beta.-Myrcene	0.495 ± 0.221	0.609 ± 0.239	0.439 ± 0.183	0.316 ± 0.137	0.428 ± 0.241	0.344 ± 0.130
d-Limonene	0.484 ± 0.176	0.508 ± 0.164	0.404 ± 0.165	0.346 ± 0.136	0.293 ± 0.050	-
Eucalyptol	7.756 ± 2.720	7.837 ± 1.355	5.687 ± 1.692	5.316 ± 1.581	6.312 ± 1.513	13.514 ± 27.260
.beta.-Ocimene	1.229 ± 0.683	1.603 ± 0.544	1.078 ± 0.367	0.608 ± 0.215	0.782 ± 0.431	1.041 ± 0.379
Cyclohexene, 3-methyl-6-(1-methylethylidene)-	0.264 ± 0.074	0.253 ± 0.076	0.233 ± 0.094	0.153 ± 0.062	0.148 ± 0.075	0.207 ± 0.077
Linalool	46.779 ± 17.978	47.924 ± 14.563	51.334 ± 11.508	58.049 ± 8.232	54.655 ± 12.337	50.441 ± 7.356
Myroxide	–	–	–	–	–	–
( +)-2-Bornanone	2.339 ± 1.115	2.258 ± 0.550	2.198 ± 0.734	2.019 ± 0.668	2.031 ± 0.532	2.105 ± 0.559
1,4-Pentadiene, 2,3,3-trimethyl-	0.020	–	–	–	0.090	0.160
Terpinen-4-ol	0.095 ± 0.036	0.120 ± 0.057	0.104 ± 0.078	0.093 ± 0.049	0.151 ± 0.172	0.162 ± 0.230
Terpineol	1.038 ± 0.409	1.122 ± 0.500	0.902 ± 0.303	0.867 ± 0.263	0.910 ± 0.316	0.800 ± 0.216
Bornyl acetate	1.332 ± 0.588	1.178 ± 0.484	1.268 ± 0.461	1.115 ± 0.384	1.095 ± 0.380	1.175 ± 0.487
1,5-Heptadiene, 2,5-dimethyl-3-methylene-	0.565 ± 0.335	0.605 ± 0.253	0.464 ± 0.168	0.526 ± 0.208	0.504 ± 0.254	0.524 ± 0.176
Eugenol	3.784 ± 5.170	5.508 ± 5.259	3.633 ± 2.997	1.538 ± 0.924	2.557 ± 2.909	3.543 ± 2.276
Phenol, 2-methoxy-4-(1-propenyl)-	0.388 ± 0.309	0.402 ± 0.184	0.378 ± 0.204	0.405 ± 0.251	0.366 ± 0.243	0.522 ± 0.203
1,11-Dodecadiyne	0.559 ± 0.455	0.432 ± 0.194	0.432 ± 0.228	0.505 ± 0.248	0.428 ± 0.226	0.510 ± 0.173
.beta.-Elemene	3.604 ± 2.026	3.238 ± 1.270	3.101 ± 1.041	3.520 ± 1.285	2.771 ± 1.056	3.189 ± 0.827
.beta.-Ylangene	0.521 ± 0.235	0.584 ± 0.197	0.563 ± 0.244	0.573 ± 0.226	0.497 ± 0.214	0.581 ± 0.186
.beta.-Cubebene	0.236 ± 0.138	0.235 ± 0.091	0.257 ± 0.116	0.260 ± 0.125	0.225 ± 0.099	0.285 ± 0.096
(Z,E)-.alpha.-Farnesene	4.779 ± 2.553	4.838 ± 3.244	5.084 ± 2.728	2.933 ± 2.210	4.103 ± 2.645	5.031 ± 2.745
Aromandendrene	0.673 ± 0.325	0.653 ± 0.352	0.548 ± 0.212	0.815 ± 0.406	0.597 ± 0.330	0.631 ± 0.205
cis-Muurola-3,5-diene						–
Humulene	0.714 ± 0.329	0.761 ± 0.251	0.615 ± 0.220	0.721 ± 0.280	0.621 ± 0.287	0.650 ± 0.206
Bicyclosesquiphellandrene	0.455 ± 0.239	0.449 ± 0.174	0.368 ± 0.147	0.392 ± 0.151	0.371 ± 0.159	0.675 ± 0.839
Germacrene D	2.298 ± 1.176	2.271 ± 0.975	2.335 ± 0.931	2.518 ± 0.960	2.098 ± 0.835	2.297 ± 0.937
gamma.-Elemene	0.698 ± 0.446	0.775 ± 0.335	0.642 ± 0.308	0.751 ± 0.306	0.696 ± 0.322	0.742 ± 0.236
alpha.-Bulnesene	1.332 ± 0.793	1.392 ± 0.506	1.267 ± 0.506	1.607 ± 0.680	1.203 ± 0.573	1.344 ± 0.401
gamma.-Muurolene	2.523 ± 1.351	2.628 ± 0.697	1.925 ± 0.660	2.246 ± 0.726	2.003 ± 0.819	2.090 ± 0.474
cis-Calamenene	0.414 ± 0.258	0.462 ± 0.152	0.451 ± 0.235	0.388 ± 0.155	0.408 ± 0.187	0.453 ± 0.140
1H-3a,7-Methanoazulene, octahydro-1,4,9,9-tetramethyl-	–	–	–	–	–	–
Cycloheptane, 4-methylene-1-methyl-2-(2-methyl-1-propen-1-yl)-1-vinyl-	0.121 ± 0.169	0.077 ± 0.042	0.151 ± 0.115	0.198 ± 0.140	0.201 ± 0.152	0.182 ± 0.197
Cubenol	1.181 ± 1.311	1.015 ± 0.374	0.880 ± 0.385	0.988 ± 0.327	0.880 ± 0.428	1.339 ± 1.190
.tau.-Cadinol	10.073 ± 10.011	7.909 ± 3.207	6.883 ± 2.035	7.530 ± 1.990	6.318 ± 2.617	6.352 ± 2.581
.alpha.-Cadinol	1.224 ± 2.613	0.441 ± 0.208	0.377 ± 0.184	0.408 ± 0.141	0.344 ± 0.182	0.377 ± 0.108
1,9-Decadiyne	–	–	–	–	–	–

Previous research has indicated that the major EO elements in basil crops are linalool, estragole, methyl cinnamate, eugenol, 1,8-cineole, methyl chavicol, geranial, and caryophyllene oxide. Other reported essential oils in basil are α-bergamotene, Ʈ-cadinol, bornyl acetate, cubenol, and γ-cadinene (Ekren et al. 2012; Vazquez et al. 2013; Sabry et al. 2019). However, the composition levels vary according to the level of nitrogen applied. Gürbüz et et al. (2009b) reported linalool at 61.10% which is almost the same percentage reported in this study. Also, Malik et al. (2011) reported that micronutrients such as Mn, Zn, and Fe play a vital role in the quality of essential oils produced from medicinal plants.

A study by Wilson et al. (2017) indicated that growing basil under a hydroponic system enhanced its EO content (eugenole) compared to the aquaponics system which is closely related to this study’s system (IAA). Moreover, Filho et al. (2018) showed that basil cultivars’ (Grecco a Palla and Toscano Folha de Alface) EO contents increased by 20.3% and 9.7%, respectively, per unit increase in salinity of the hydroponic solution. However, Burduca et al. (2019) investigated wastewater from recirculating aquaculture systems on basil essential oil composition. It was observed that plants irrigated with fish wastewater contained high amounts of β-linalool (+ 11%), eugenol (+ 1, 5%), eucalyptol (− 12%), methyl eugenol (− 1.9%), germacrene D (+ 22%), and α-trans-bergamotene (− 4.7%). Another study by Sabry et al. (2019) investigated the performance of different basil cultivars grown in the open field under Egyptian conditions. It was observed that the EO yield of all cultivars varied between 0.15 and 0.46% and linalool was the main EO component (37.25–61.99%) followed by 1,8 cineole (9.5–23.6%), α-bergamotene (2.87–14.18%), and Ʈ-cadinol (2.13–8.3%) respectively. Ekren et al. (2012) also reported an increase in EO with of purple basil with deficit irrigation at 50% field capacity (EO ratio 1.10%).

## Conclusion

The study shows the feasibility of integrating the four different systems, i.e., solar energy, desalination, aquaculture, and agriculture production. This is the first time such a study of an integrated desalination, aquaculture, and agriculture production using renewable energy has been investigated. This pilot study revealed the following main findings: RO desalination recorded a permeate recovery of 22%, salt rejection of 98.7%, and permeate salinity of 200–300 ppm. Tilapia recorded survival rate was 97.7% and a mean harvest weight of 458 g at a productivity of 25 kg/m^3^ of water. Fish effluents are a rich source of fertilizers to support crop growth; it recorded a significant biomass yield of sweet basil compared to irrigation with chemical fertilizers. Foliar application (Ca, Mg, Zn, Mn, and Fe) increased the vegetative growth of sweet basil irrespective of the treatment. Effluent irrigation plus supplemental foliar fertilizers recorded maximum plant height of 62 cm and highest yield of 14.7 ton/ha and gave a basil productivity of 5 kg/m^3^ of effluents. GC–MS analysis showed that linalool was the main constituent with the highest composition, followed by tau.-cadinol, eucalyptol, eugenol, (Z,E)-.alpha.-farnesene, and beta.-elemene. Therefore, these results suggest that irrigating sweet basil with fish effluents and supplementing it with foliar fertilizers can result in an increased crop herbage yield, and maximum oil content. Commercial application of such a model on larger scale could be a potential solution to sustain and reduce freshwater usage in agriculture, increase water productivity, and increase unit area production.

## Data Availability

All data generated or analyzed during this study available from Mendeley data at http://dx.doi.org/10.17632/492nxb2yk3.1 with an embargo period but are available from the corresponding author on reasonable request.
